# HERVs establish a distinct molecular subtype in stage II/III colorectal cancer with poor outcome

**DOI:** 10.1038/s41525-021-00177-w

**Published:** 2021-02-15

**Authors:** Mahdi Golkaram, Michael L. Salmans, Shannon Kaplan, Raakhee Vijayaraghavan, Marta Martins, Nafeesa Khan, Cassandra Garbutt, Aaron Wise, Joyee Yao, Sandra Casimiro, Catarina Abreu, Daniela Macedo, Ana Lúcia Costa, Cecília Alvim, André Mansinho, Pedro Filipe, Pedro Marques da Costa, Afonso Fernandes, Paula Borralho, Cristina Ferreira, Fernando Aldeia, João Malaquias, Jim Godsey, Alex So, Traci Pawlowski, Luis Costa, Shile Zhang, Li Liu

**Affiliations:** 1 Illumina Inc., San Diego, CA USA; 2 Instituto de Medicina Molecular - João Lobo Antunes, Faculdade de Medicina, Universidade de Lisboa, Lisbon, Portugal; 3 Centro Hospitalar Universitário Lisboa Norte, Hospital de Santa Maria, Lisbon, Portugal; 4 Faculdade de Medicina, Universidade de Lisboa, Lisbon, Portugal

**Keywords:** Cancer, Gastrointestinal cancer

## Abstract

Colorectal cancer (CRC) is one of the most lethal malignancies. The extreme heterogeneity in survival rate is driving the need for new prognostic biomarkers. Human endogenous retroviruses (hERVs) have been suggested to influence tumor progression, oncogenesis and elicit an immune response. We examined multiple next-generation sequencing (NGS)-derived biomarkers in 114 CRC patients with paired whole-exome and whole-transcriptome sequencing (WES and WTS, respectively). First, we demonstrate that the median expression of hERVs can serve as a potential biomarker for prognosis, relapse, and resistance to chemotherapy in stage II and III CRC. We show that hERV expression and CD8+ tumor-infiltrating T-lymphocytes (TILs) synergistically stratify overall and relapse-free survival (OS and RFS): the median OS of the CD8-/hERV+ subgroup was 29.8 months compared with 37.5 months for other subgroups (HR = 4.4, log-rank *P* < 0.001). Combing NGS-based biomarkers (hERV/CD8 status) with clinicopathological factors provided a better prediction of patient survival compared to clinicopathological factors alone. Moreover, we explored the association between genomic and transcriptomic features of tumors with high hERV expression and establish this subtype as distinct from previously described consensus molecular subtypes of CRC. Overall, our results underscore a previously unknown role for hERVs in leading to a more aggressive subtype of CRC.

## Introduction

Colorectal cancer represents one of the most commonly diagnosed cancers worldwide and the second leading cause of cancer death in western countries^1^. While the clinicopathological (CP) features such as tumor grade and stage, tumor-node-metastasis (TNM) staging, lymph node (LN) involvement (pN0-pN2) are well-established biomarkers of poor prognosis^2,3^, the significance of molecular and cellular markers is not well demonstrated in a clinical setting. Among several genomic features of CRC, *RAS* mutations represent the first biomarker integrated into clinical practice for CRC for negative predictive response to *EGFR* targeted therapy^4^. Likewise, patients with *BRAF* mutations exhibit a poor prognosis^4^, while *PIK3CA* and *PTEN* mutant CRC patients develop resistance to first-line chemotherapy and anti-PD-1 and anti-CTLA-4 antibody-based immunotherapies, respectively^5,6^. Lynch syndrome, which is caused by a mutation in the *MLH1*, *MSH2, MSH6*, or *PMS2* genes, greatly increases the risk of colorectal and endometrial cancers^7^. Recently, genome- and exome-wide biomarkers have been introduced, with microsatellite instability (MSI) as the first pan-cancer biomarker for response to immune checkpoint inhibitor (ICI)^8,9^. In addition, recent studies have established gene expression-based CRC classifications as robust predictors of clinical outcome^10,11^.

HERVs compose a family of retrotransposons constituting about 1–8% of the human genome^12^. HERVs possess similar genomic structure to exogenous retroviruses such as human immunodeficiency virus (HIV) and human T-cell leukemia virus (HTLV), and are composed of gag, pol, and env regions between two long terminal repeats (LTRs)^12^. While representing footprints of previous viral infections, most hERVs are transcriptionally inactive; however, several recent studies have demonstrated that epigenomic modification can reactivate this family of genes^13^. There have been several functions attributed to hERVs such as regulating the development of human embryonic stem cells^14^ and involvement in a wide variety of infections^15,16^, autoimmune disease^17^, and carcinogenesis through transactivation of proto-oncogenes^18–21^. Several models have been proposed to explain hERV mediated cancer progression suggesting hERVs are potentially oncogenic, although the causative role of hERVs in cancer is controversial^20^. Thus, we wanted to determine whether hERVs are implicated in CRC, and if so, elucidate the utility of hERVs as a potential biomarker of clinical outcome.

We used whole-exome and whole-transcriptome sequencing (WES/WTS) to evaluate 114 patients with CRC. We determined that hERV expression defined a previously unknown molecular subtype of CRC. We established that this novel molecular subtype was independent of previously characterized consensus molecular subtypes (CMS) of CRC. Finally, we show that hERV expression in combination with absolute CD8+ TIL concentration can predict prognosis, relapse, and resistance to chemotherapy more effectively than previously proposed correlates of CRC outcome. In cancer cells, active hERV expression may promote an innate immune response that enables immuno-therapy for treatment^20^.

## Results

### Study design

In total, 114 patients with stage II and III CRC were selected with an equal proportion of microsatellite instability high/stable (MSI-H/MSS) tumors as measured with MSI-PCR (see “Methods”). Table 1 summarizes the CP characteristics of the cohort. All patients were diagnosed with CRC, followed at the Oncology Division of Hospital de Santa Maria, Lisbon, and treated as per institutional clinical practice in accordance to international guidelines, namely ESMO and NCCN guidelines^22–24^. We performed WES for paired tumor and normal tissues as well as WTS on the tumor tissue for all patients (see “Methods” and Supplementary Data 1 and 2). One patient was excluded due to the low alignment rate of WTS reads.

**Table 1:** Cohort summary.

MSI		MSH	MSS
Number of patients		56	57
Median age (years)		74.5	66
Stage II:		33	35
Adjuvant chemo^a^		7/33	7/35
Stage III:		23	22
Adjuvant chemo^b^		14/23	14/22
Right side		48	25
Left side		7	26
Sidedness	Right	Left	Other
Number of patients	73	33	7
Stage	II	III	Other
Number of patients	68	45	0

^a^The total number of stage II patients who received adjuvant chemotherapy compared to the total number of patients in each MSI group.

^b^The total number of stage III patients who received adjuvant chemotherapy compared to the total number of patients in each MSI group.

We first compiled a list of hERVs and retrotransposon associated sequences from previous published studies^25,26^, and built a customized reference by appending the new reference to the human genome (hg19) reference built as previously described^25^. Next, we bioinformatically quantified the expression of different hERV sequences by aligning WTS reads to the new reference (see “Methods” and Supplementary Data 3). The results illustrated that hERVs are constitutively expressed in most CRC tumor tissues, with some patient tumor tissues exhibiting relatively higher expression of hERVs (Supplementary Fig. 1). HERV expression was confirmed by digital PCR on a subset of hERVs with variable expression across the patient cohort (Supplementary Fig. 2).

### Impact of hERV expression on immunophenotype landscape

**Fig. 1:**
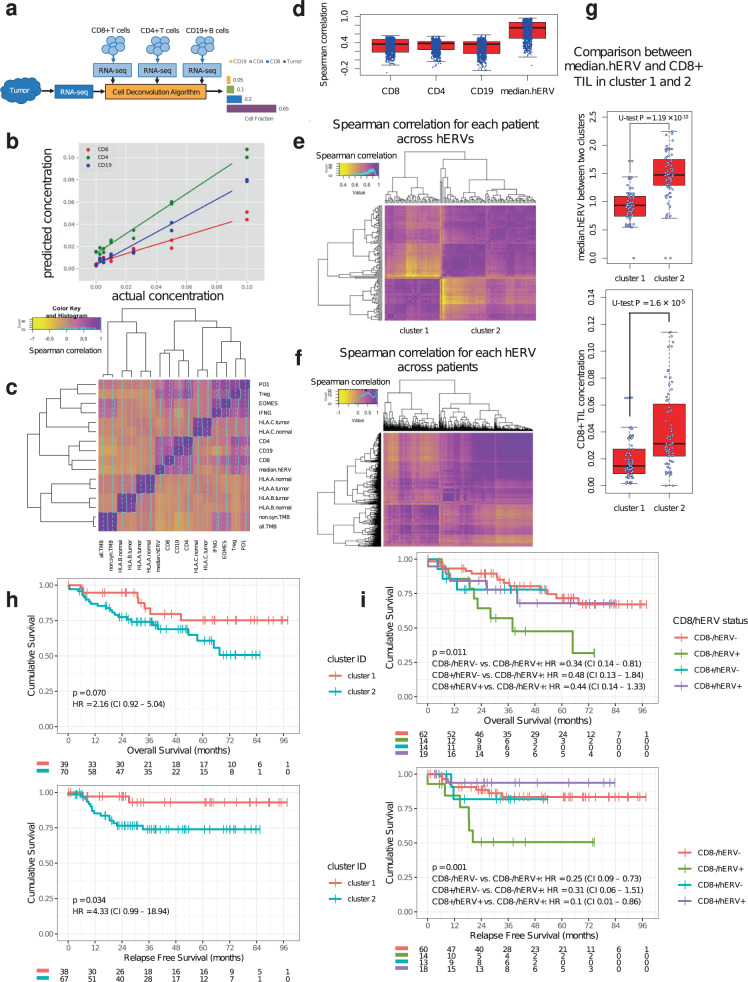
Immune characteristic and prognostic power of hERVs in CRC. **a** Schematic of immune cell deconvolution using FRICTION. **b** Immune cell input titration shows high accuracy of FRICTION in predicting CD8+, CD4+, and CD19+ immune cells using WTS data from fresh frozen tissues. **c** Heatmap illustrates the Spearman correlation between different immune related features. TILs are closely related to hERV expression demonstrating the immunogenicity of hERVs. High association between CD8+ T cells, Treg and PD1 indicates exhaustion CD8+ T cells in most patients. **d** Correlation of each individual hERVs transcripts with TILs and median.hERV. **e** Spearman correlation for each patient across hERVs. **f** Spearman correlation of hERVs across patients. **g** Two distinct classes of hERVs are associated with low and high median.hERV and CD8+ TIL. **h** Kaplan–Meyer curves of OS and RFS of patients stratified based on clusters obtained from hierarchical clustering in (**e**). cluster 1 (i.e. median.hERV^low^) demonstrates a significantly better outcome compared to cluster 2 (i.e. median.hERV^high^). **i** Kaplan–Meier curves of OS and RFS for each group with respect to CD8 + and median.hERV status (log-rank *P* values are shown). The black lines in the “middle” of the boxes are the median values for each group. The vertical size of the boxes illustrates the interquartile range (IQR). Whiskers represent 1.5 IQR.

Previous studies reported strong immunogenicity of hERVs both in vitro and in vivo^27–30^. To profile TIL pattern of CRC and potential association between hERV expression and TILs, we developed a novel immune cell-deconvolution method, Fractional Recovery of Immune Cell Types in Oncology NGS (FRICTION, see “Methods” and Fig. 1a). In contrast to Newman et al.^31^, but similar to Racle et al.^32^, we focused on the deconvolution of absolute cell fraction. This is enabled by our gene selection procedure, as well as our feature normalization that places each of our cell-type signatures on the same scale. We validated FRICTION on several tumor types (including colon) using immune cell input titration (Supplementary Fig. 3 and Fig. 1b). Overall, we showed that FRICTION is able to accurately estimate immune cell infiltration using WTS of bulk tumor tissues. We applied FRICTION to the WTS data we generated for this cohort, enabling accurate measurement of absolute concentrations of CD8+, CD4+, and CD19+ TILs (Supplementary Data 4 and 5). Most hERVs showed a high association with TILs (Fig. 1c, d) suggesting hERVs confer immunogenicity, confirming previous findings^27–30^. As expected, tumor purity and immune infiltration were inversely correlated (Spearman correlation = −0.25, *P* = 0.0087). However, median hERV was not correlated with tumor purity (Spearman correlation = 0.07, *P* = 0.47). Moreover, there was a high correlation between hERVs within each patient (Fig. 1e) as well as between patients for each hERV (Fig. 1f), indicating high similarity in their expression profile and regulation. Interestingly, hierarchical clustering pinpointed two prominent clusters of patients based on hERVs implying potential distinct phenotypes (Fig. 1e, f). While none of the CP factors showed significant over-representation in either of these clusters, there was a significant difference in the median expression of all hERVs (median.hERV) between the two clusters (Mann–Whitney U-test, *P* = 1.19 × 10^−10^). As expected, higher median.hERV was accompanied by higher CD8+ T-cell infiltration (Mann–Whitney U-test, *P* = 1.6 × 10^−5^) (Fig. 1g). We also explored whether MSI status could predict lymphocyte infiltration. MSI-H patients had significantly higher activated immune cell signatures such as CTL and effector T-cell score, but not absolute CD8+ T-cell concentration (Supplementary Fig. 4). In addition, MSI, TMB, and HLA diversity scores^33^ were independent of immune or hERV-related features (Fig. 1c and Supplementary Data 6).

### Assessment of hERVs as a potential biomarker in CRC

**Fig. 2:**
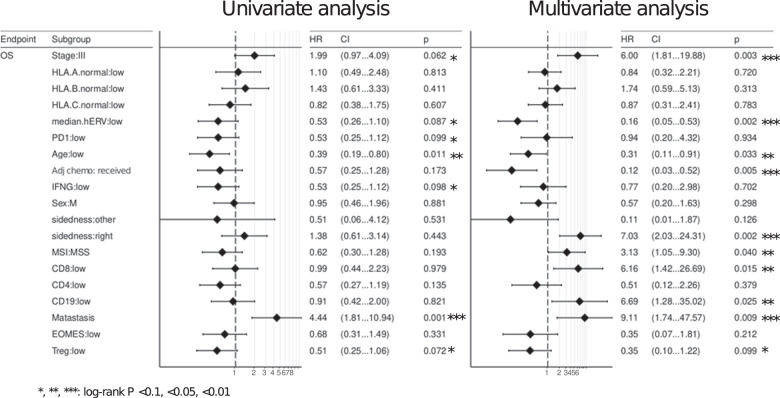
Univariate and multivariate survival analysis. OS overall survival.

Next we looked at disease outcome. The two classes identified by unsupervised hierarchical clustering strongly stratified patients’ survival in a univariate analysis. This suggests that the cluster with higher median.hERV entails worse prognosis (Fig. 1h, OS-RFS log-rank *P* = 0.070–0.034, HR = 2.16–4.33). Hence, we hypothesized hERV expression is a potential biomarker in CRC. As shown in Fig. 1f, the majority of hERV loci are co-expressed and therefore, median.hERV is highly correlated with general hERV expression throughout the genome. Previous studies^34,35^ have highlighted the expression of young HERV-H loci in the course of colorectal carcinoma. Although a more specialized panel of hERV loci may render a higher discriminatory power compared a genome-wise approach such as median hERV expression (Supplementary Fig. 5), selection of only a subset of hERVs is less robust, more sensitive to measurement noise, might be patient specific, and requires larger cohorts to avoid overfitting and rendering reproducibility in separate validation cohorts. Besides, loci specific activation might vary between cancer types. On the contrary, median hERV, analogous to other genome-wide markers such as TMB and MSI, can potentially serve as a pan-cancer biomarker for patient selection (this remains to be explored in the future studies). As median.hERV is a robust measure of hERV activation per tumor tissue, it can be utilized as a representative biomarker (see “Methods”). To demonstrate the utility of median.hERV, we compared the predictive power of median.hERV with other previously described biomarkers. We assessed the impact of other CP, genomics and transcriptomics factors on patients’ survival by discretizing continuous features using a top 30% threshold of the distribution per factor (Supplementary Fig. 5). Notably, we evaluated several potential biomarkers recently proposed to be predictive of response to ICIs, to evaluate whether they can also stratify patients’ response to adjuvant chemotherapy. Potential biomarkers evaluated included expression of FOXP3 (or Treg gene signature), IFNG (or activated TIL signatures), MSI, PD(L)1^36^, and HLA diversity score^33^. Several factors showed a strong predictive power in both univariate and multivariate analysis including stage, metastasis status, MSI, adjuvant treatment, sidedness, but not ICI-specific biomarkers as expected. Interestingly, both CD8+ and median.hERV were strong predictors of clinical outcome in a multivariate analysis (Fig. 2). We performed similar univariate survival analysis for each specific hERV and illustrated that most hERVs were capable of stratifying patients’ overall survival (OS) (HR ranging from 0.25 to >1); most hERVs were associated with a poor prognosis (Supplementary Fig. 6a). Median age of MSI-H and MSS patients were 74.5 and 66 years old, respectively. Due to the correlation between age and MSI status, we also have included age as a confounder in our multi-factorial survival analysis model which as expected, demonstrated a poor clinical outcome in older patients. However, we did not see a significant correlation between age and median.hERV (Pearson *R* = −0.12, *P* = 0.18) or CD8+ level (Pearson *R* = 0.1, *P* = 0.2). The age difference between CD8+ (median age = 69.5 years) and CD8− (median age = 71 years) patients was negligible. We observed patients with higher median.hERV are mainly observed in younger patients (median age = 67.5 years) compared to older patients (median age = 73 years old).

**Fig. 3:**
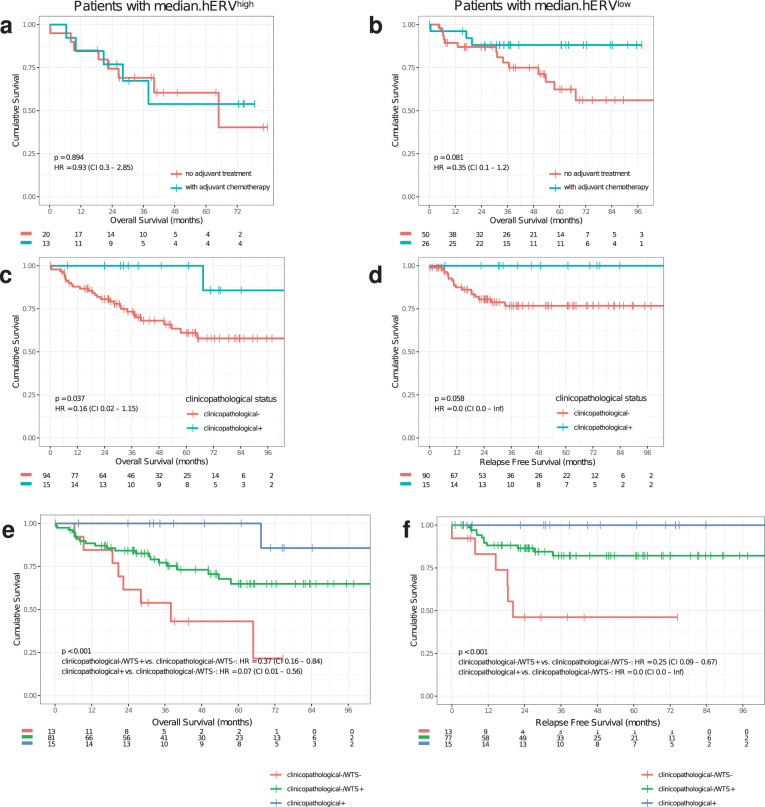
Clinical relevance of hERV and CD8+ TIL biomarkers. Kaplan–Meier curves of OS and RFS for each group are shown. **a**, **b** Patients with high median.hERV develop resistance to chemotherapy. **c**, **d** Clinicopathological factors can predict survival outcome. clinicopathological− is defined as patients with unfavorable age (top 30%), or stage (III), or sidedness status (right). **e**, **f** Incorporating median.hERV and CD8+ biomarkers can further stratify patients with poor clinical outcome (log-rank *P* values are shown). CD8−/hERV+ subgroup is labeled as WTS− as opposed to WTS+ which composes CD8+/hERV+, CD8−/hERV−, and CD8+/hERV− subgroups.

Previous studies reported patients with higher CD8+ TILs have favorable prognosis^37^, and similar observations were obtained in the multivariate analysis of this cohort (Fig. 2). Our analysis also showed a positive association between CD19+ TILs and survival in a multivariate analysis. However, since CD8+ TIL concentration was a stronger predictor of survival, we focused on this cell type. Inspired by immunogenicity of hERVs, we hypothesized patients with low CD8+ TILs but high hERV expression (CD8−/hERV+) would constitute a subtype with poor prognosis. Indeed, we observed a strong stratification with this subgroup in terms of both OS and RFS (see “Methods” for OS and RFS calculations) such that the median OS (RFS) of CD8−/hERV+ subgroup is 29.8 (19.7) months compared with 37.5 (32.8) months for other subgroups (HR = 4.4, log-rank *P* < 0.001). This suggests that the CD8+ TIL concentration and hERV expression can be used synergistically to predict clinical outcome (Fig. 1i and Supplementary Fig. 6b). Furthermore, after stratifying patients by high or low median.hERV comparison of patients who received adjuvant chemotherapy or no adjuvant treatment, revealed a strong benefit of adjuvant chemotherapy only in patients with low median.hERV (Fig. 3a, b). Importantly, this suggests that hERV can induce resistance to adjuvant chemotherapy.

### Association between hERVs and consensus molecular subtypes

It is worth noting that despite several lines of evidence supporting that genomic and transcriptomic biomarkers have predictive and prognostic power, none of these signatures are currently employed in routine clinical use in the context of CRC^3^. As previously shown in this study, age, stage, and sidedness could guide clinicians for therapy selection and avoid over-treatment. For example, Fig. 3c, d exhibit patients with favorable CP factors—defined as clinicopathological+ (i.e. young patients with stage II left sided/rectal CRC), could be treated differently than others (OS log-rank *P* = 0.037, HR = 0.16). However, CD8−/hERV+ biomarker can further stratify the subgroup with poor clinical outcome, i.e. clinicopathological- (OS log-rank *P* < 0.001, HR = 0.07, Fig. 3e, f). In Fig. 3e, f, the CD8−/hERV+ subgroup is labeled as WTS− as opposed to WTS+ which composes CD8+/hERV+, CD8−/hERV−, and CD8+/hERV− subgroups.

**Fig. 4:**
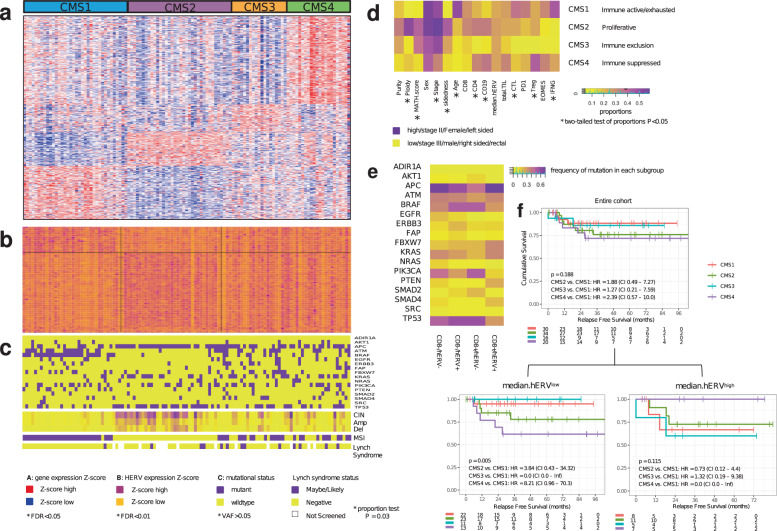
HERVs establish a distinct molecular subtype in CRC. **a** CMS classification defines CMS1-4 for patients in this cohort. **b** hERVs are abundant in all CMSs. Heatmaps in **a** and **b** represent Z-scores for genes with FDR < 0.05 and FDR < 0.01. **c** Mutational and copy number profile, MSI and LS status for each CMS observed in this cohort. **d** Enrichment analysis portraits clinicopathological and immuno-phenotypical characteristic of each CMS. **e** Heatmap shows the frequency of each gene mutations per CD8/hERV subgroup. **f** Kaplan–Meier curves of RFS for each group are shown. Incorporation of median.hERV into CMS classification boosts the predictive power CMS (log-rank *P* values are shown).

To this end, an international consortium that included a panel of expert research groups proposed a CMS classification to facilitate the clinical translation of colorectal cancer subtyping^10^. Hence, we sought to elucidate if the CD8−/hERV+ subtype was within a specific previously known CMS. Using the pretrained random forest model^38^, we classified each patient into CMS1-4 based on gene expression profile of tumor samples: 6 out of 113 patients could not be classified into any class, while others were uniquely classified. FDR < 0.05, Fig. 4a. Note that since the 113 patients enrolled in this study were selected to attain an equal MSI/MSS ratio, the proportions labeled as CMS1-4 differ from other studies that are representative of CRC. By performing one-versus-others differential gene expression analysis (U-test FDR < 0.01, Fig. 4b), we observed several hERVs to be over-expressed in CMS1 and 2 but not 3 and 4. Nevertheless, we could measure the expression of hERVs across all subtypes CMS1-4.

In parallel, we performed WES of all 113 tumors to explore the genomic characterization of each CMS. Mutational profile analysis showed (Fig. 4c) enrichment of *APC*^*mut*^, *TP53*^*mut*^, and *KRAS*^*mut*^ in CMS2-4 but not CMS1. Conversely, *ATM*^*mut*^ and *BRAF*^*mut*^ were over-represented in CMS1 but not in CMS2-4. The frequency of each driver mutation for this cohort is shown in Supplementary Fig. 7a. We also investigated the frequency of arm-level deletion and amplification events (Chromosomal Instability, CIN) and observed CMS2 is strikingly associated with CIN^high^ while CMS3–4 show CIN^medium^ and CMS1 is CIN^stable^ (Fig. 4c and Supplementary Fig. 7b, c). As expected, 18q and 17p loss of heterozygosity (LOH) were common across patients. Likewise, we detected a similar pattern for gene deletion and amplification events occurring primarily in CMS2 (Supplementary Fig. 6d, e). Previous studies suggested CMS1 and CMS2 to be associated with MSI-H and MSS phenotypes while CMS3–4 consist of a mixed MSS/MSI-H phenotype^10^. We determined the MSI status of each patient using WES and confirmed previous findings (Fig. 4c). The high frequency of *BRAF* p.V600E mutation in CMS1 points to a sporadic MSI-H phenotype. As a result, we hypothesized this CMS1 MSI-H phenotype differs from CMS3–4.

Following NCCN guidelines^39^ (Supplementary Fig. 8), we developed an algorithm to determine whether CMS3–4 are germline driven and associated with Lynch Syndrome (LS) phenotype. We identified the LS status of patients by performing ultra-deep sequencing of tumor samples on TruSight™ Oncology 500 (TSO500)—a research target enrichment sequencing assay that enables comprehensive genomic profiling and measures tumor mutation burden (TMB) and microsatellite instability (MSI) (Fig. 4c, Supplementary Data 7). Notably, we classified 13 patients to have potential Lynch syndrome. Interestingly, we observed a strong enrichment of LS in CMS3/4 subtypes suggesting that MSI-H patients in CMS3/4 form a distinct type of MSI-H from CMS1 (proportion test, *P* = 0.03). Note that LS patients in CMS2 subtype are all MSS while all patients with a positive Lynch syndrome status in CMS3/4 are also MSI-H suggesting that MSI-H phenotype in CMS3/4 could be primarily driven by Lynch syndrome and tend to be associated with germline mutations than the sporadic mutations observed in elderlies with CMS1 of CRC. LS status predicted from tumor only sequencing was in high agreement with matched normal sequencing to confirm germline variants, with a positive percent agreement (PPA) of 100% (95% CI: 75.3–100%) and a negative percent agreement (NPA) of 98.2% (95% CI: 93.6–99.8%). LS has also been attributed to favorable survival outcome^40^. Thus, we asked whether CMS3/4 patients should further be subcategorized with respect to the LS status of patients. Within CMS3/4 patients, survival analysis showed patients with LS have a favorable prognosis compared with others (Supplementary Fig. 9a, b); this effect was not statistically significant, which was attributed to the low number of LS patients (OS: HR = 0.3, CI 0.04–2.35, *P* = 0.22).

Comparison of biomarker status between different analysis approaches revealed that MSI status of all patients matched between MSI-PCR, WES, and TSO500. Similarly, TMB estimations were highly concordant between TSO500 and WES (Supplementary Fig. 10). All patients with a high TMB were also MSI-H with the exception of one MSS patient with a POLE mutation. This rare mutation has previously been ascribed to a defective DNA repair mechanism, which explains high TMB phenotype and can be associated with a better clinical outcome^41^ and favorable response to ICI^42^. In addition, using WTS, we found three patients with actionable fusions, one *NTRK1-LMNA* (CMS1), one *ALK-EML4* (CMS1), and one *BRAF-ZFP64* (CMS4). A *RSPO2-MATN2* fusion, a potential drug target, was also detected in one CMS1 patient. Taken together, we further define genomic features of each CMS and highlight the value of paired whole exome (or targeted panel) with transcriptome sequencing in facilitating clinical guidance.

Each CMS has been attributed to distinct pathological groups^10^. Enrichment analysis (Fig. 4d) confirmed previous findings that CMS1 is related to immune active (high IFNG and CTL scores) while CMS4 is immune suppressed (high Treg score measured by *FOXP3* expression). We observed enrichment of CMS1 in older patients which could be explained by the sporadic MSI-H phenotype in CMS1. In addition, CMS1 was over-represented in right-sided CRC patients in contrast to left-sidedness of CMS2. CMS4 was associated with stage III and younger CRC patients. Finally, CMS2 showed the highest ploidy and with CMS3 represented the highest intra-tumoral heterogeneity as measured by mutant-allele tumor heterogeneity (MATH score)^43^. However, none of the analyzed genomic features showed an enrichment in median.hERV^high/low^ groups suggesting that hERV activation is an event independent of known cancer driver mutations and possibly associated with epigenomic modification^13^ (Fig. 4d and e).

**Fig. 5:**
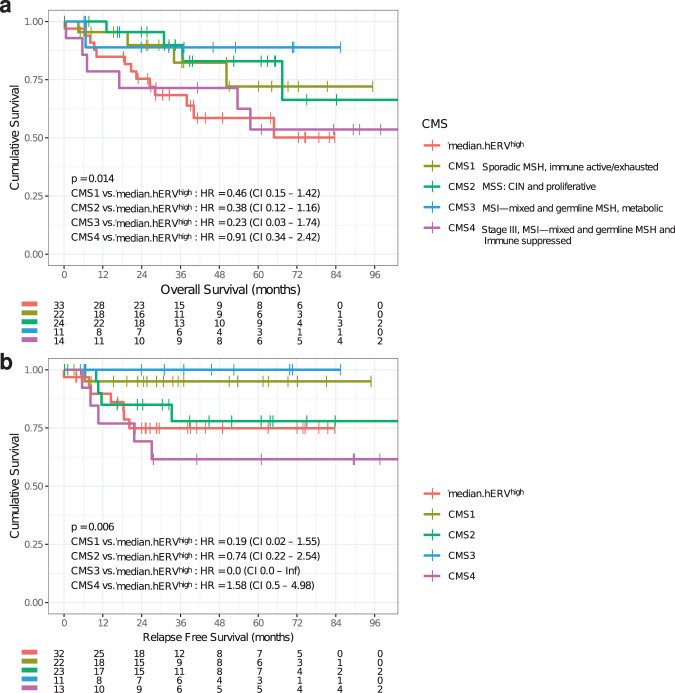
New proposed CMS classification. **a**, **b** Kaplan–Meier curves of OS and RFS for each group are shown. CMS4 and median.hERV^high^ independently indicate different groups with poor prognosis (log-rank *P* values are shown).

Other studies used CMS classification to predict CRC-related survival and showed that CMS4 confers the worst outcome which was confirmed in our cohort even though this stratification did not reach a statistical significance (OS-RFS: log-rank *P* = 0.2–0.19 > 0.1). However, incorporating median.hERV^high^ subtyping dramatically improved CMS classification suggesting that the median.hERV^high^ is an independent molecular subtype and can confound previous CMS classifications (Fig. 4f and Supplementary Fig. 11). The lack of enrichment of median.hERV^high^ subtype in any of CMSs (Fig. 4d) further recognized median.hERV^high^ subtype as an important previously unappreciated CRC subtype. All in all, we demonstrated median.hERV^high^ subtype together with CMS4 have the worst survival in terms of both OS and RFS (OS-RFS: log-rank *P* = 0.014–0.006, Fig. 5a, b). Besides, exploratory analysis of previous trials such as FIRE-3 trial (first-line therapy with FOLFIRI^44^ plus either cetuximab or bevacizumab in 592 KRAS exon 2 wild-type metastatic CRC patients) and CALGB/SWOG 80405^45^ (a phase III trial that compared the addition of bevacizumab or cetuximab to infusional fluorouracil, leucovorin, and oxaliplatin or fluorouracil, leucovorin, and irinotecan as first-line treatment of advanced CRC), showed CMS classification is prognostic for mCRC; however, at present time has no direct impact on clinical decision-making and may need further refinement. Therefore, we propose the addition of median.hERV^high^ subtype of CRC to CMS can facilitate clinical decision-making and improve CMS classification for patient selection.

## Discussion

In this study, we introduced hERVs as a potential biomarker for survival, relapse, and resistance to adjuvant chemotherapy and showed CRC patients with high median.hERV can succumb to metastatic disease. Moreover, our results illustrated that hERV expression can identify a novel, previously overlooked CMS which together with CMS1-4 can improve CRC classification. Whether the expression of hERVs is a cause or consequence of oncogenesis is out of the scope of this study. We demonstrated that hERV expression can be utilized as a potential biomarker of poor prognosis and can also inform physicians of unfavorable responses to adjuvant chemotherapy prior to treatment. Previous studies have shown the importance of hERV expression as a potential biomarker in anti-PD1 antibody-based ICI adjuvant treatment of clear cell renal cell carcinoma^25^. The expression of hERVs have been reported across pan-cancers^46^, and immunogenicity of hERVs has been well-documented both in vitro and in vivo^27–30^. Here, we also showed patients expressing high amounts of hERV might not be responsive to chemotherapy and exhibit poor clinical outcome. These patients could benefit from ICIs since blocking immune checkpoint molecules can reinvigorate the immune system targeting hERV expressing tumor cells. Interestingly, we observed a high correlation between median.hERV and several checkpoint molecules including *PD(L)1*, *CTLA4*, *TIM3*, and *LAG3* (Supplementary Data 8). Currently, ICI is the approved treatment for patients with unstable microsatellite status (MSI-H); however, MSI-H status encompasses only roughly 15% of CRC patients. Our study suggests that 30% of MSS patients would potentially benefit from ICI or epigenetic-based therapies alone or in combination with ICI. This percentage increases when a less stringent hERV^high^ threshold is set. The CpG island methylator phenotype (CIMP) status can further accommodate therapeutic selection, specifically for epigenetic-based therapies; however, we did not have CIMP status of the patients in this cohort due to the unavailability of the patient methylation data. Nevertheless, previous studies^10^ showed a significant association between CMS1 and CMS3 with CIMP-high and CIMP-low while CIMP-negative phenotype is mainly enriched in CMS2 and CMS4. Therefore, using our WTS data generated and the obtained CMS classifications, we can estimate CIMP status of each patient. The potential benefit of such therapeutics must be further explored in future studies. Future studies can further evaluate hERV as a biomarker for ICI treatment selection across pan-cancers by evaluating patients’ response to ICI in non-MSI-H and not Pol-mutated patients.

## Methods

### Cohort characteristics

We pseudo-randomized a cohort composed of 114 patients with stage II/III colorectal cancer such that CP factors with significant impact on survival outcome (e.g., treatment, stage, sex, and MSI status) are balanced. Previous studies^47^ have shown several proposed biomarkers are confounded by other clinicopathological factors, e.g., MSI status, the stage and the sex of patients. Therefore, it is necessary to balance these factors to account for confounders. Although our cohort characteristic might not be representative of other observed CRC cohorts, pseudo-randomization is a statistically sound approach to balance potential confounders. Also note that due to the low frequency of MSI-H occurrence in CRC (~15%), we would have missed potential associations of our discovered biomarkers with MSI status which further motivated us to select a larger fraction of MSI-H patients in this study. One patient (CR698) was excluded due to low WTS alignment rate (*N* = 113). Cases were staged according to The American Joint Committee on Cancer (AJCC) staging system, 8th edition^48^. In the entire cohort, median (interquartile range [IQR]) age was 71 (69–76) years; 70 patients were female; and 68/114 (59.5%) and 45/114 (39.5%) patients were diagnosed with stage II and III CRC according to AJCC, respectively. Patients had not previously received neoadjuvant chemo or radiotherapy (only 2 rectal cases were included in this cohort). A total of 17/45 (37.8%) stage III patients did not receive adjuvant chemotherapy (11 were above 70 years) and 14/68 (20.6%) stage II patients received adjuvant chemotherapy. Selected chemotherapy regimens included FOLFOX or Capecitabine^22,23^. Stage II patients have indication for adjuvant chemotherapy if additional risk factors are present, whereas for stage III patients, adjuvant chemotherapy is the standard-of-care bellow the age of 70 unless other comorbidities preclude this indication. The study was approved by the ethics committee from Hospital de Santa Maria, Centro Hospitalar Universitário Lisboa Norte (Lisbon, Portugal) and all patients provided signed informed consent.

### Survival analysis

We defined relapse-free survival (RFS) and overall survival (OS) as follows:

For RFS, survival time: A-B

For OS survival time: A-C

where,

A: cutoff date for follow-up or censoring date, or study end point date (i.e., death/relapse date or last follow-up)

B: date of surgery

C: date of diagnosis

*P* values for Kaplan–Meier analyses were derived using the log-rank test. The hazard ratios for survival analysis were calculated by a univariate or multivariate Cox proportional hazards model in R.

### Sample extraction

CRC (*n* = 114) and paired normal colon tissues (*n* = 114) were collected by a Medical Pathologist from surgically removed specimens. Normal samples were taken from adjacent tissue more than 2 cm away from the tumor. Tissues were embedded in optimal cutting temperature (OCT) medium, snapshot frozen in liquid nitrogen within 40 min of collection and preserved at −80 °C. For each sample, DNA and RNA were extracted from three cryosections 30-μm thick, using the AllPrep DNA/RNA Micro Kit (Qiagen), following the manufacturer’s protocol. Presence of CRC cells in tumor samples and absence in normal tissues were confirmed by H&E staining after tissue collection and sectioning by a Medical Pathologist.

### DNA/RNA quality control

Extracted DNA was quantified using the Qubit dsDNA High-Sensitivity. DNA quality was determined by the delta-Cq method using the Illumina TruSeq™ FFPE DNA Library Prep QC Kit. Samples were required to have delta-Cq<6 for library preparation. Extracted RNA was quantified with Qubit RNA High Sensitivity (Thermo Fisher Scientific). RNA quality was determined by the DV200 method using Agilent RNA 6000 Pico Kit; samples were required to have a DV200 > 40% for library preparation.

### Microsatellite instability testing by PCR

MSI status was determined using the Promega MSI Analysis System, Version 1.2 (PN MD1641). Two nanograms input DNA was used for each PCR reaction, and K562 High Molecular Weight DNA was used as positive control. PCR was done on the GeneAmp PCR System 9700 Thermal Cycler using 9600 emulation mode with the cycling profile according to the user manual (Thermo Fisher Scientific).

The Applied Biosystems 3130xl Genetic Analyzer (Thermo Fisher Scientific) was used to detect amplified fragments. One microliter of each amplified sample was used for input according to manufacturer recommended specifications. Data were analyzed with Applied Biosystems GeneMapper Software 5. For each tumor-normal pair, five microsatellite markers were compared. Presence of new alleles in the tumor sample that were absent in the normal tissue indicated MSI. Tumor samples in which two or more markers were altered were classified as MSI-High.

Promega’s MSI Analysis System is comprised of seven markers, which are co-amplified using fluorescently labeled primers. There are five mononucleotide repeat markers used for MSI determination (BAT-25, BAT-25, NR-21, NR-24, and MONO-27), and two pentanucleotide repeat markers (Penta C and Penta D) used to determine mismatched samples or contamination.

### Whole-transcriptome sequencing

Illumina TruSeq Stranded Total RNA with 100 ng input RNA per sample was used for generating whole-transcriptome libraries following the manufacturer’s protocol. IDT for Illumina TruSeq RNA UD Indexes (96 indexes) was used for sample indexing. Libraries were quantified with Qubit dsDNA High Sensitivity assay (Thermo Fisher Scientific) and normalized for sequencing on Illumina NovaSeq™ 6000 S2 (36-plex) or S4 (72-plex) flow cell with 76 bp paired-end sequencing targeting ~200 million read pairs per sample. On average, each sample yielded 303 million reads and 26,394 transcripts identified (Supplementary Data 1).

### Exome sequencing

Illumina Nextera™ Flex for Enrichment with 40 ng input DNA per sample was used for generating matched tumor and normal exome-enriched libraries, with the following optimizations. IDT for Illumina Nextera DNA Unique Dual Index Set A was used for sample indexing with 9 cycle of indexing PCR. Samples were quantified with Qubit dsDNA High Sensitivity assay and four libraries were pooled for enrichment (4-plex) such that 500 ng of each library was used for a total of 2000 ng per enrichment pool. Target enrichment was performed using IDT xGen Exome Research Panel (4 µl per enrichment reaction). A single hybridization was done overnight at 58 °C, with 12 cycles of post-enrichment PCR. Libraries were quantified by Qubit dsDNA High Sensitivity assay, normalized and pooled. Samples were sequenced (12-plex per lane) as 151 bp paired-end reads on the NovaSeq 6000 S4 flow cell using the XP workflow for individual lane loading. On average, each sample yielded 602 million reads and MEDIAN_TARGET_COVERAGE depth of 476X (Supplementary Data 2).

### TruSight Oncology 500

Illumina TruSight Oncology 500 was used for generating tumor DNA libraries to determine TMB, MSI, and Lynch syndrome status. Forty ng input of tumor DNA was used, and libraries were prepared and enriched according to manufacturer’s instructions recommendations, with 8-plex library pooling for 101 bp paired-end sequencing on the NextSeq™ 550 sequencing system. Assay sequencing analysis metrics are summarized in Supplementary Data 7.

### Gene expression and fusion calling

Raw reads were aligned using STAR-2.6.1 to hg19 human reference genome using the following options: --outSAMtype ‘BAM’ ‘SortedByCoordinate’ --outSAMattributes ‘NH’ ‘NM’ ‘MD’–outSAMmapqUnique ‘50’ --peOverlapNbasesMin 10 --peOverlapMMp 0.1 --outSAMtlen 2 --outSAMstrandField ‘intronMotif’ --outFilterType ‘BySJout’ --outSJfilterCountUniqueMin ‘-1’ ‘2’ ‘2’ ‘2’ --outSJfilterCountTotalMin ‘-1’ ‘2’ ‘2’ ‘2’ --outFilterIntronMotifs ‘RemoveNoncanonical’ --chimSegmentMin ‘12’ --chimJunctionOverhangMin ‘12’ --chimScoreDropMax ‘20’ --chimSegmentReadGapMax ‘5’ --chimScoreSeparation ‘5’ --chimScoreJunctionNonGTAG ‘-100’ --chimOutType ‘WithinBAM’ options. We used rsem-1.2.31 for gene quantification. Transcript per million (TPM) normalized values were used for gene expression analysis. Fusion calling was performed using manta-1.3.2 after removing duplicate aligned reads.

### hERV quantification

We mined the literature and combined a set of hERV and cancer-related retrotransposon sequences to build a costume reference file of 3200 retrotransposon/hERV-related sequences as described by previous studies^25,26^. We used salmon-0.11.3 for hERV transcript quantification after alignment of raw reads by STAR aligner --outFilterMultimapNmax 10 --outFilterMismatchNmax 7. Raw quantified transcripts were normalized by dividing the total number of counted transcripts by the library size for each sample times million (counts per million or CPM). We defined 0.5 CPM as the noise threshold for each transcript and set the CPM < 0.5 values to zero to reduce the influence of noise on the downstream analysis. Moreover, any genes with median CPM < 0.1 across all tumor samples were considered as not expressed and removed from the downstream analysis. The resulting 831 unique hERV transcripts were used for the rest of the analysis. We defined median.hERV as the median of the CPM of the 831 hERVs transcripts for each sample. We have performed a robustness study by downsampling the number of studied hERVs to 80% of the originally considered loci and demonstrated that median.hERV is robust across 100 rounds of this experiment (Supplementary Fig. 12).

### Confirmation of hERV quantification by ddPCR

Four hERVs with a range of high, medium, and low median hERV expression were selected to confirm the WTS expression with ddPCR. Eight samples from the cohort were selected to cover a range of high to low hERV expression. NONO expression was used as a reference gene for calculating relative gene expression in WTS and ddPCR quantitation. ddPCR assays were designed by Bio-Rad for the following genes: hERV_3351 (Unique AssayID: dHsaCNS314897488), hERV_2180 (dHsaCNS612174471), hERV_1073 (dHsaCNS443134067), hERV_2256 (hERV-E-env dHsaCNS701378478), and NONO (dHsaCPE5025872, dHsaCPE5025873). TruSeq Stranded Total RNA cDNA synthesis was performed with 50 ng total RNA. cDNA was diluted to a reaction concentration of 1 ng per reaction for droplet generation and PCR of one hERV probe and NONO reference probe in triplicate reactions using Bio-Rad ddPCR Supermix for Probes (No dUTP). Droplets were read with the Bio-Rad QX200 Droplet Reader. Spearman correlation and *p* values were calculated for each hERV.

### Immune cell deconvolution using FRICTION

FRICTION uses a support vector regression-based technique to estimate the fraction of RNA from a set of input cell types. To perform deconvolution, we require a set of signatures from some number of cell types of interest. Then, given the profile of an unknown mixed sample, we predict the fraction of the unknown sample that can be explained by the given signatures. FRICTION focuses on the selection and normalization of genes in ways that promote the detection of absolute cell fraction (i.e., the percentage of total cells) in contrast to other methods that focus on relative cell fraction (i.e., the percentage of immune cells) or statistical enrichment. First, a set of gene signatures are developed. We have created gene signatures using a set of purified cells as well as an explicit set of background samples from a variety of tissue types. In contrast to Newman et al.^31^, but similar to Racle et al.^32^, we focus on the deconvolution of absolute cell fraction. This is enabled by our gene selection procedure, as well as our feature normalization that places each of our cell-type signatures on the same scale.

### Validation of FRICTION

Methods for the FRICTION algorithm development and validation are described in So et al.^49^. Briefly, RNA purified from CD4+, CD8+, and CD19+ immune cells were titrated into RNA extracted from fresh frozen normal colon, kidney, pancreas, ovary, rectum, uterus, esophagus, thyroid, and bladder tissues. Libraries were prepared with TruSeq RNA Exome for sequencing and immune cell quantitation by FRICTION. In addition, cryopreserved melanoma tumors were used for quantification of immune cells through flow cytometry or RNA sequencing analyzed by FRICTION. These methods demonstrate a linearity in quantifying these cells with a median *R*^2^ > 0.98.

### Consensus molecular subtyping

In order to classify 113 patients into one of the previously defined CMSs, we used CMScaller 0.99.1 package in R^38^ with default parameters and RSEM expected count data as input. One hundred and eight tumor samples were assigned to a unique CMS with FDR < 0.05.

### Variant calling and tumor mutational burden

We performed DNA alignment using the Burrows-Wheeler Aligner (BWA-MEM) algorithm with the Sequence Alignment/Map (SAMtools) utility to align DNA sequences in FASTQ files to the hg19 genome. The total number of somatic mutations identified was normalized to the exonic coverage in megabases as TMB. Somatic variant calling was performed using Strelka-2.9.10^50^ on paired tumor-normal BAM files for each sample after removing duplicate reads. Low confident SNVs were removed using: Tumor VAF≥0.05, DP.tumor≥50, DP.normal≥20, AD.tumor≥5, VAFnormal/VAFtumor<0.2. Only variants called on both strands are called as high confident.

### Copy number alteration

CNV robust analysis for tumors (CRAFT) was used to investigate gene deletion and amplification in tumor samples^51^. Briefly, CRAFT takes as input a set of baseline samples and determines the read coverage of each amplicon, or “bin” for the sample. Then, a sample’s bin count is modeled as a linear combination of baselines, and the linear model prediction is used as a baseline-corrected value. GC quantile normalization removes the effects of GC bias on the baseline-corrected values. After normalization, gene amplification or deletion events are determined using empirically determined cutoff values. We defined the probability of deletion or amplification at arm level as the total number of deleted or amplified genes divided by the total number of genes. We considered an arm to be deleted or amplified if the probability of deletion or amplification exceeds 20%.

### Microsatellite instability

Microsatellite sites were defined as noncoding homopolymers with a repeat size of 10–50 bases. Sites with an ethnicity bias or lower coverage on the panel were excluded from further analysis. Unstable microsatellite sites were detected by assessing the shift in the distribution of the length of a microsatellite site in a tumor sample against the paired normal sample for WES pipeline or baseline (48 normal tissue samples) for TSO500 MSI pipeline. A site was identified as unstable when it is outside the known range. The MSI score of each tumor sample was calculated as the number of unstable sites divided by the total number of assessed sites. Tumor samples were classified as MSI-H if MSI score ≥threshold, where threshold = 20% and 30% for TSO500 and WES pipelines, respectively. We used 130 and 3713 MSI sites, comprising homopolymers with ≥10 bp repeat for TSO500 and WES MSI pipelines, respectively.

### Tumor purity, ploidy, and intra-tumoral heterogeneity

We estimated tumor purity and ploidy using Sequenza 2.1^52^ using paired tumor-normal WES BAM files after removing duplicate reads. To determine intra-tumoral heterogeneity, we calculated the Mutant-Allele Tumor Heterogeneity (MATH) score^43^ by sciClone 1.1^53^ on normalized coverage data together with estimated tumor purity and high-quality SNVs.

### Statistics

R version 3.5.1 was used for data postprocessing and rendering figures. The black lines in the “middle” of the boxes are the median values for each group. The vertical size of the boxes illustrates the interquartile range (IQR). Whiskers represent 1.5 IQR.

### Reporting summary

Further information on research design is available in the Nature Research Reporting Summary linked to this article.

## Supplementary information

Supplementary Information

Supplementary Data 1

Supplementary Data 2

Supplementary Data 3

Supplementary Data 4

Supplementary Data 5

Supplementary Data 6

Supplementary Data 7

Supplementary Data 8

Supplementary Data 9

Reporting Summary

## Data Availability

All data associated with this study are present in the main paper or the Supplementary Materials. All genomic and transcriptomic correlates of CRC prognosis are available in Supplementary Data 9. The raw data used in this study have been deposited in the NCBI Database available under accession number PRJNA689313.

## Code availability

Scripts used in data analysis for this manuscript can be found at GitHub: https://github.com/mgolkaram/HERVs-establish-a-distinct-molecular-subtype-in-stage-II-III-colorectal-cancer-with-poor-outcome/tree/master.
