# Supplementary material for: HERVs establish a distinct molecular subtype in stage II/III colorectal cancer with poor outcome
Source: NPJ Genom Med. 2021 Feb 15;6:13. doi: 10.1038/s41525-021-00177-w (PMC7884730; doi:10.1038/s41525-021-00177-w)
Supplement: Supplementary file 1 — Supplementary Information [file 41525_2021_177_MOESM1_ESM.pdf]

Supplementary Fig. 1

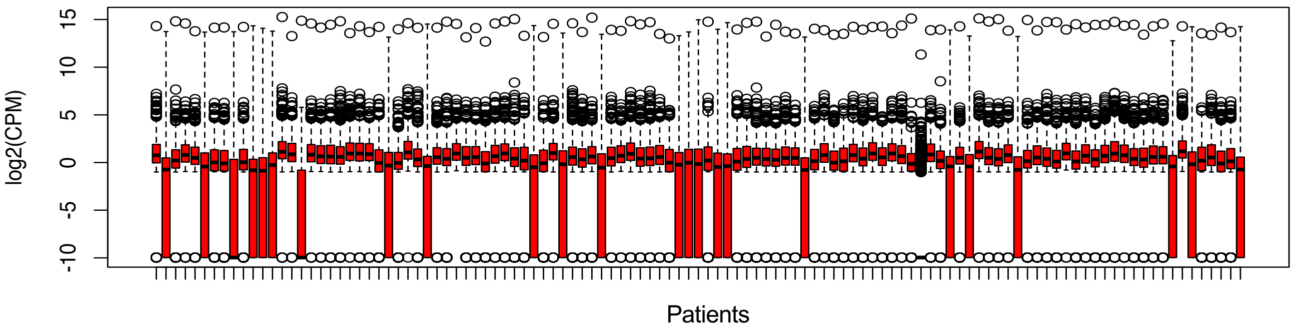

Distribution of normalized hERV expression across patients. hERV is constitutively expressed across patients with over-expression in some compared to others.

Supplementary Fig. 2

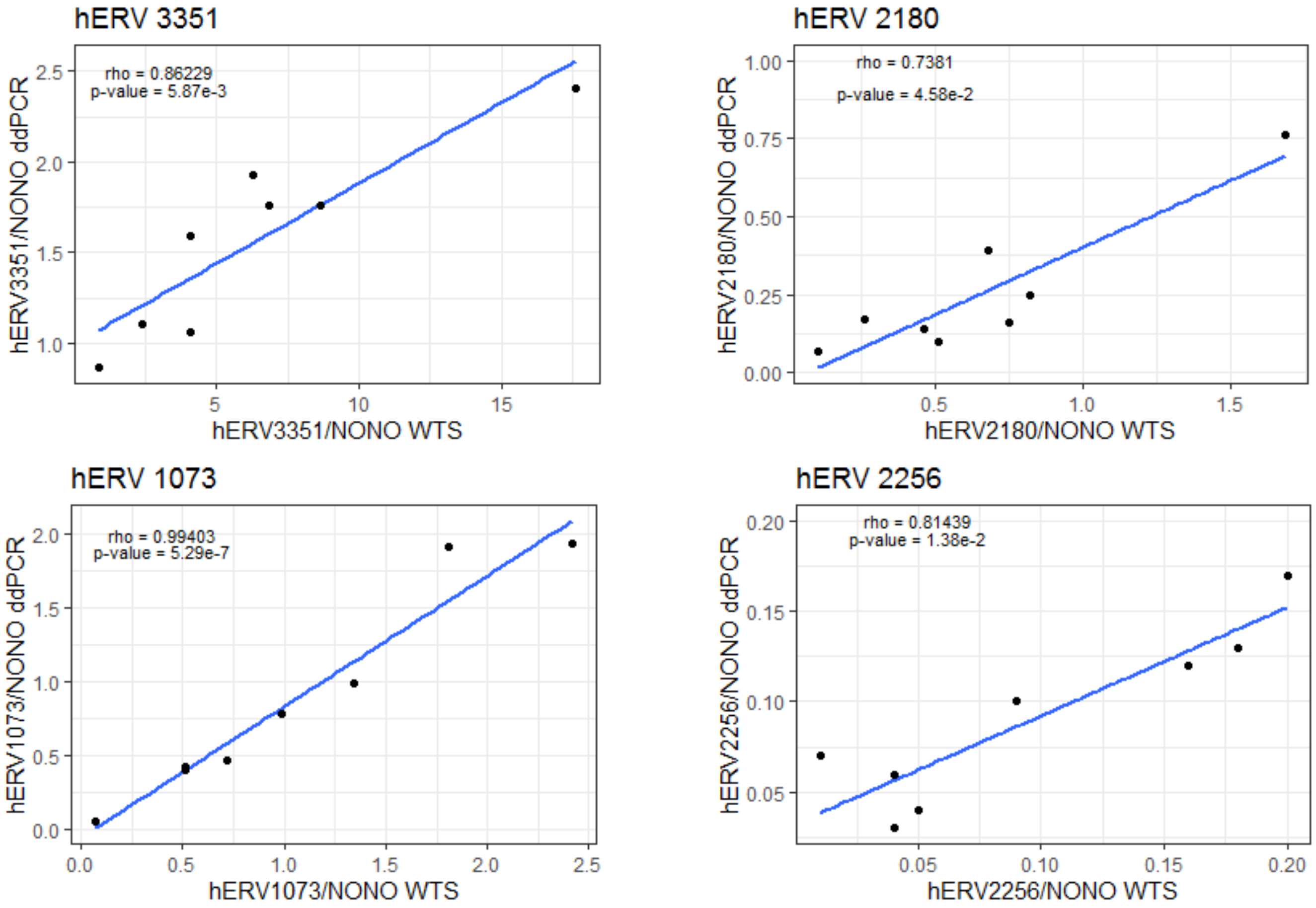

Sample	WTS Relative Expression				ddPCR Relative Expression			
	3351/NONO	2180/NONO	1073/NONO	2256/NONO	3351/NONO	2180/NONO	1073/NONO	2256/NONO
CR719	0.87	0.10	0.07	0.01	0.87	0.07	0.06	0.07
CR725	4.08	0.51	0.72	0.05	1.06	0.10	0.47	0.04
CR735	2.39	0.26	0.51	0.04	1.10	0.17	0.43	0.06
CR366	8.67	0.82	1.81	0.20	1.76	0.25	1.92	0.17
CR355	4.08	0.46	0.51	0.04	1.59	0.14	0.41	0.03
CR798	6.83	0.75	1.34	0.18	1.76	0.16	0.99	0.13
CR245	6.31	0.68	0.99	0.09	1.93	0.39	0.78	0.10
CR407	17.63	1.68	2.42	0.16	2.40	0.76	1.94	0.12

Expression of hERVs confirmed by ddPCR. Four hERVs were selected for confirmation of expression by ddPCR. HERV expression relative to NONO gene expression was confirmed in 8 samples ranging from low to high expression in the cohort. Spearman rho and p-values confirm significant correlation between whole transcriptome and ddPCR relative expression.

Supplementary Fig. 3

R^2 values		FRICTION		
	% Titration	CD8	CD4	CD19
Colon #1	0-5%	0.97	0.96	0.95
Colon #2	0-5%	0.96	0.96	0.98
Colon #3	0-5%	0.88	0.80	0.77
Colon #4	0-5%	0.94	0.96	0.98
Kidney	0-10%	0.94	0.99	0.97
Pancreas	0-10%	0.88	0.97	0.99
Ovary	0-10%	0.87	0.99	0.97
Rectum	0-10%	0.97	0.99	0.99
Uterus	0-5%	0.93	0.98	0.99
Esophagus	0-5%	0.90	0.99	0.97
Thyroid	0-5%	0.93	0.98	0.99
Bladder	0-5%	0.60	0.98	0.97

FRICTION benchmarking results. Immune cell deconvolution predictions are in good agreement with the percentage titration of immune cells across several cancer types. Colon #1-4 are four different colon tumor samples.

Supplementary Fig. 4

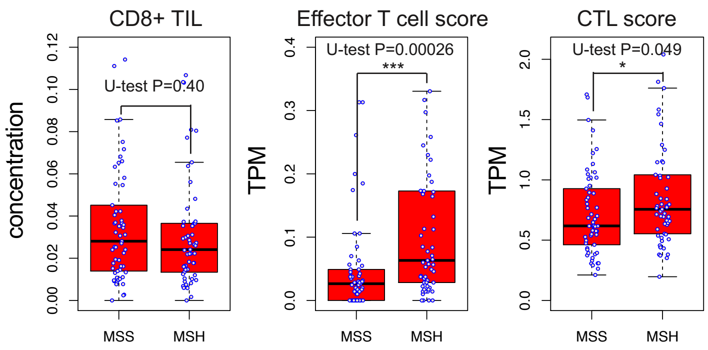

Association between MSI and immune activity. MSI status was associated with CTL score ($\sqrt{PRF \times GZMB}$) and effector T cell score ($\sqrt{EOMES \times \gamma IFN}$) but not CD8+ concentrations.

Supplementary Fig. 5

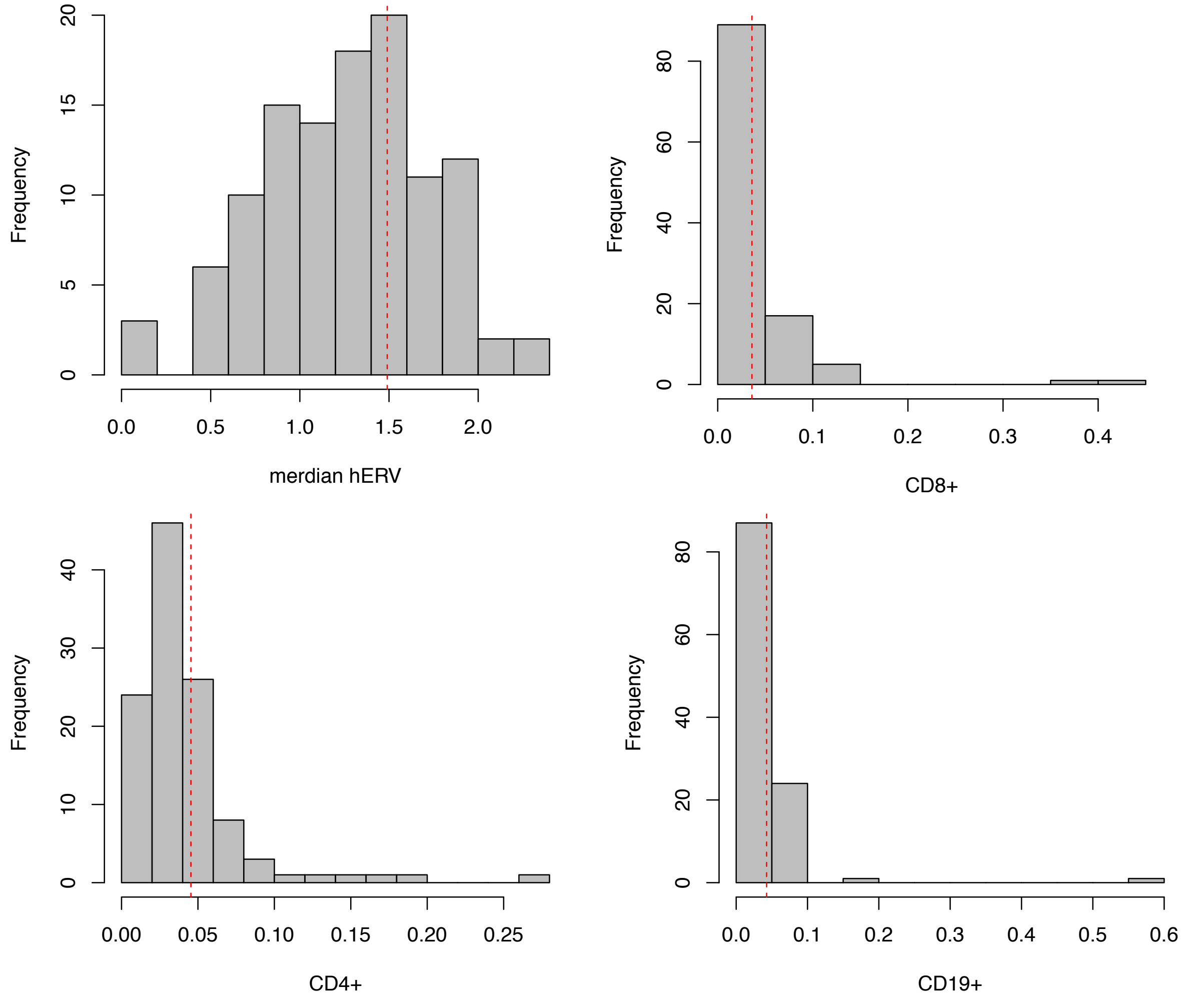

Discretization of continuous biomarkers. We set the threshold as the top 30% of the distribution of each variable. Patients with higher than the threshold values are defined as high and vice versa.

Supplementary Fig. 6

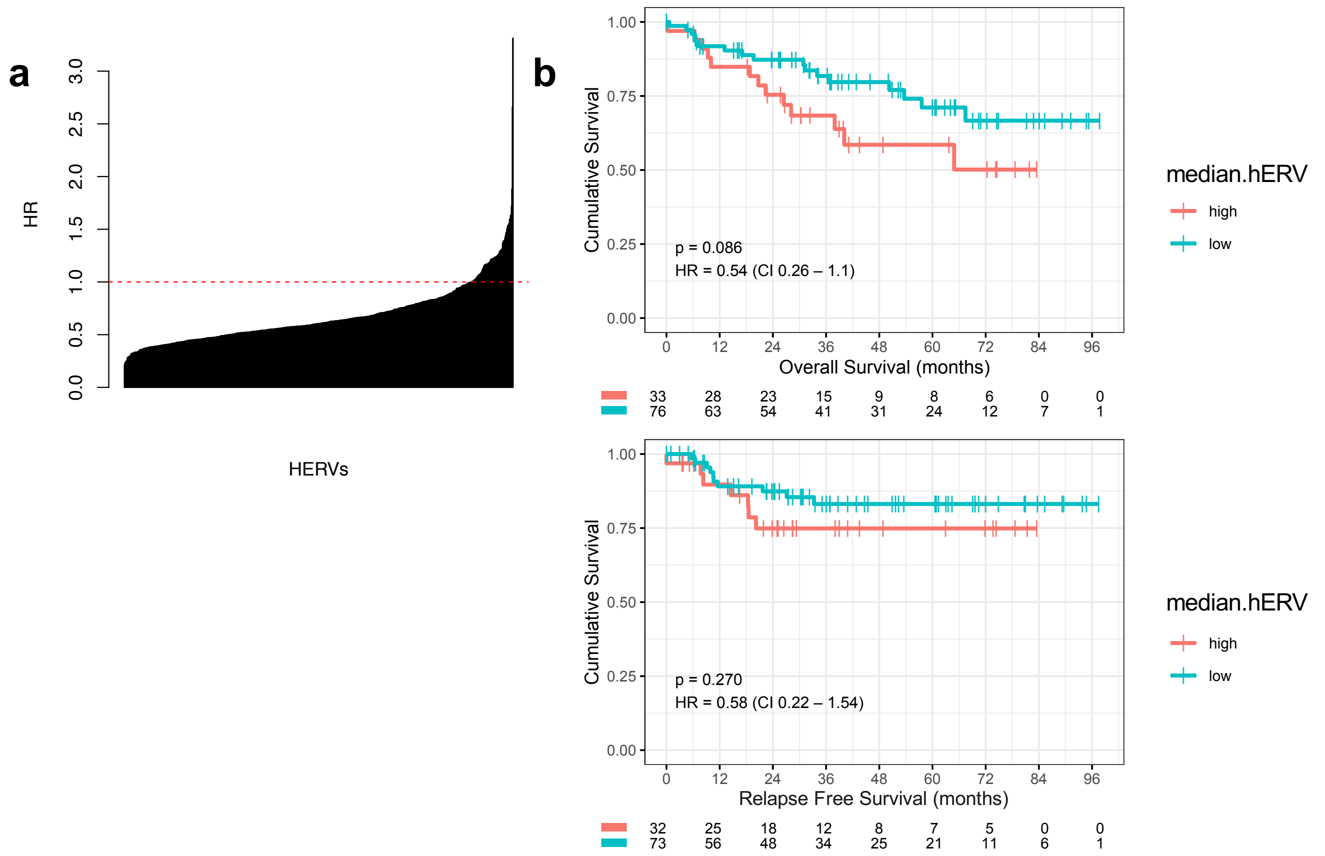

Distribution HRs of each hERV using OS as end point. a, Most hERVs are capable of stratifying patients' prognosis. **b,** Kaplan-Meier curves of OS and RFS median.hERV^{high} and median.hERV^{low} groups.

Supplementary Fig. 7

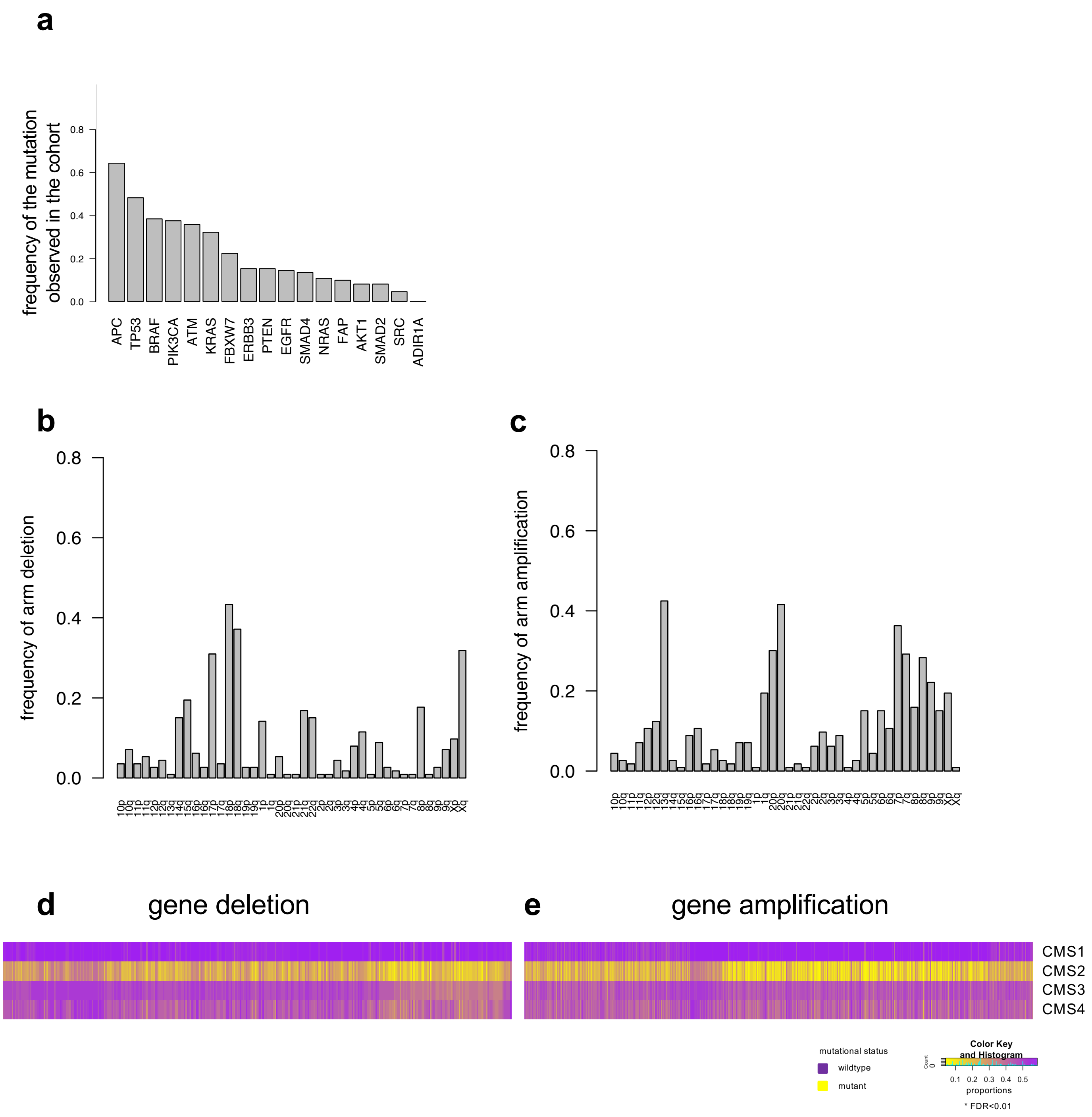

Summary of genomic features of the cohort. a, frequency of driver mutations. **b** and **c**, frequency of arm deletions and amplifications. **d** and **e**, enrichment of gene deletion and amplification events of CRC patients in each CMS.

Supplementary Fig. 8

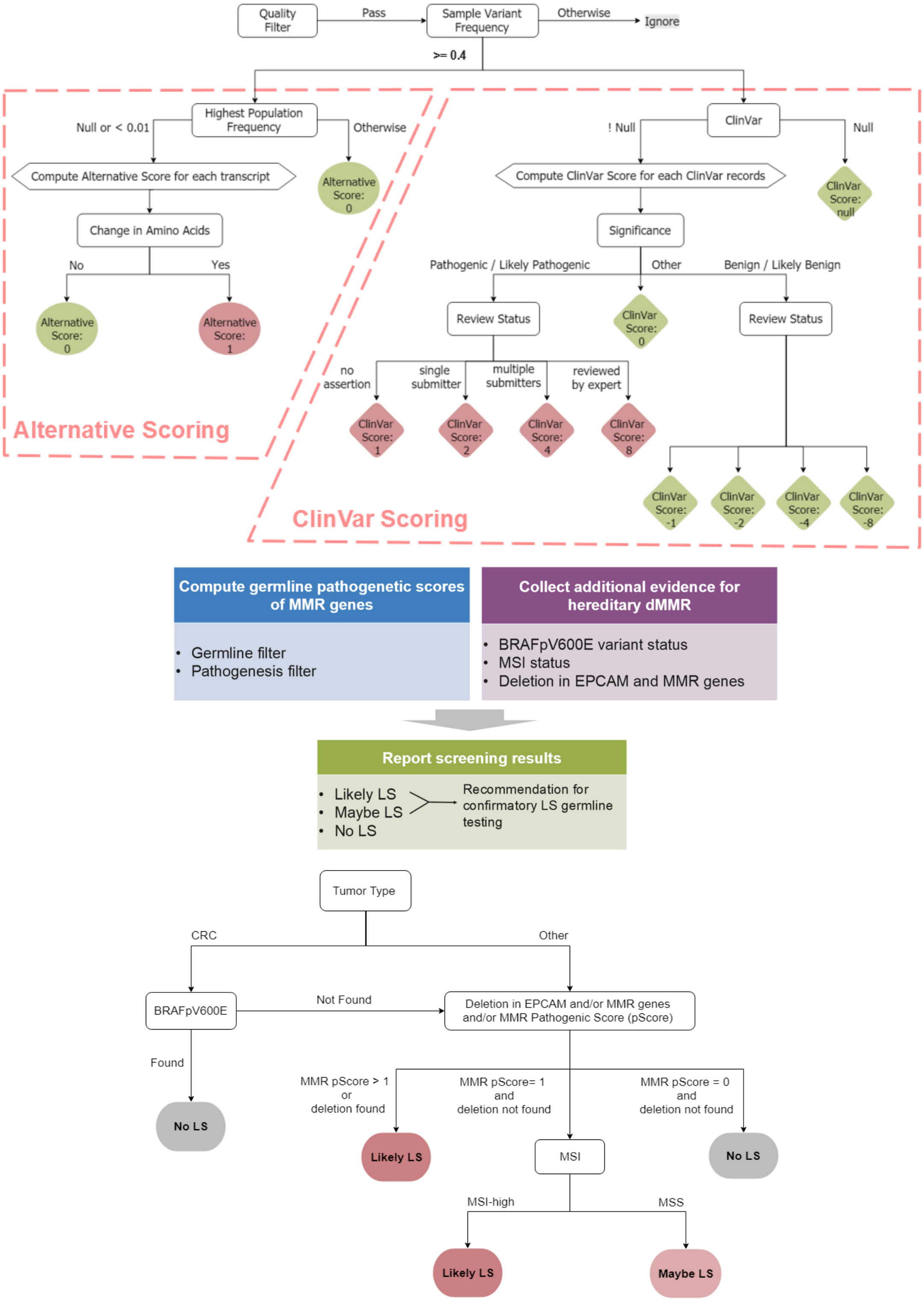

Lynch syndrome identification algorithm using TSO500 assay.

Supplementary Fig. 9

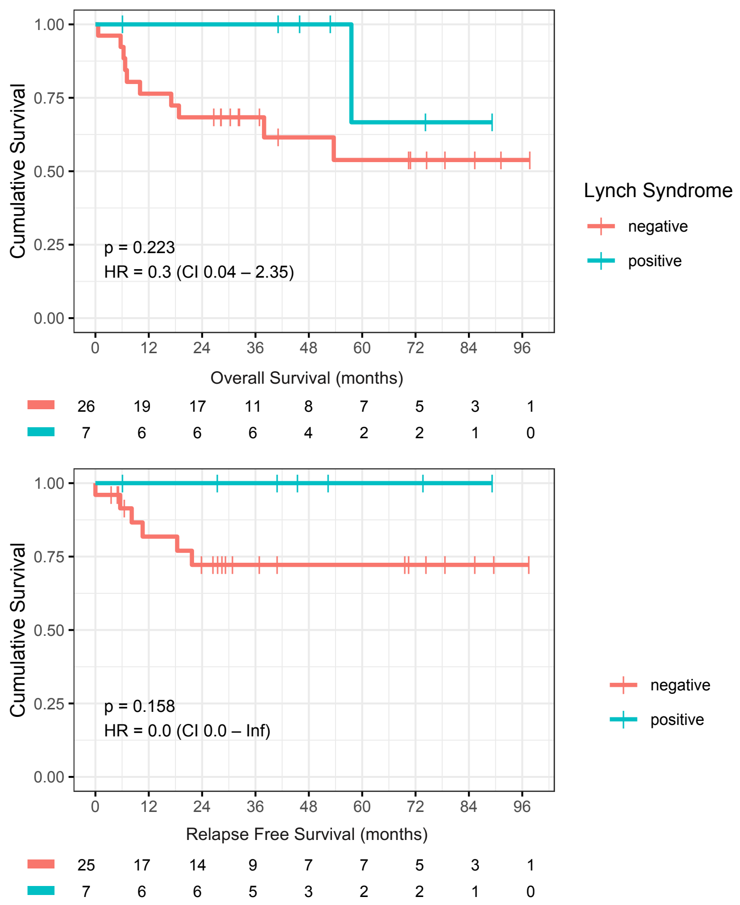

Survival analysis using Lynch syndrome status as the predictive variable. Patients with LS tend to confer favorable response compared to others. End points: OS and RFS.

Supplementary Fig. 10

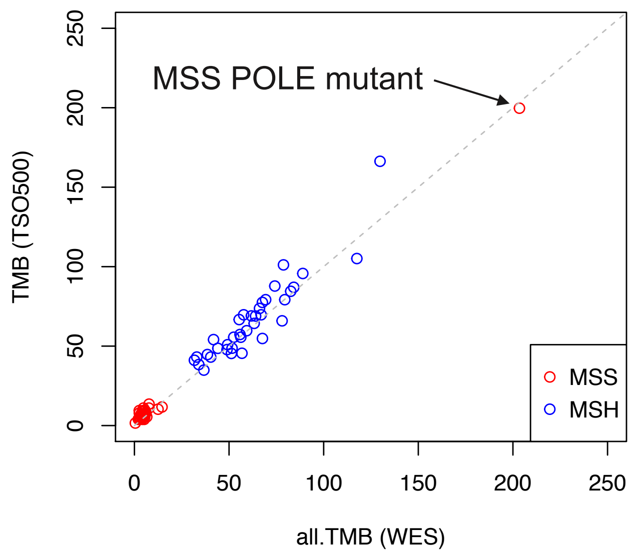

High concordance between TMB and MSI measurements through TSO500 and WES. With the exception of one POLE mutant patient, all MSS patients had low TMB and vice versa.

Supplementary Fig. 11

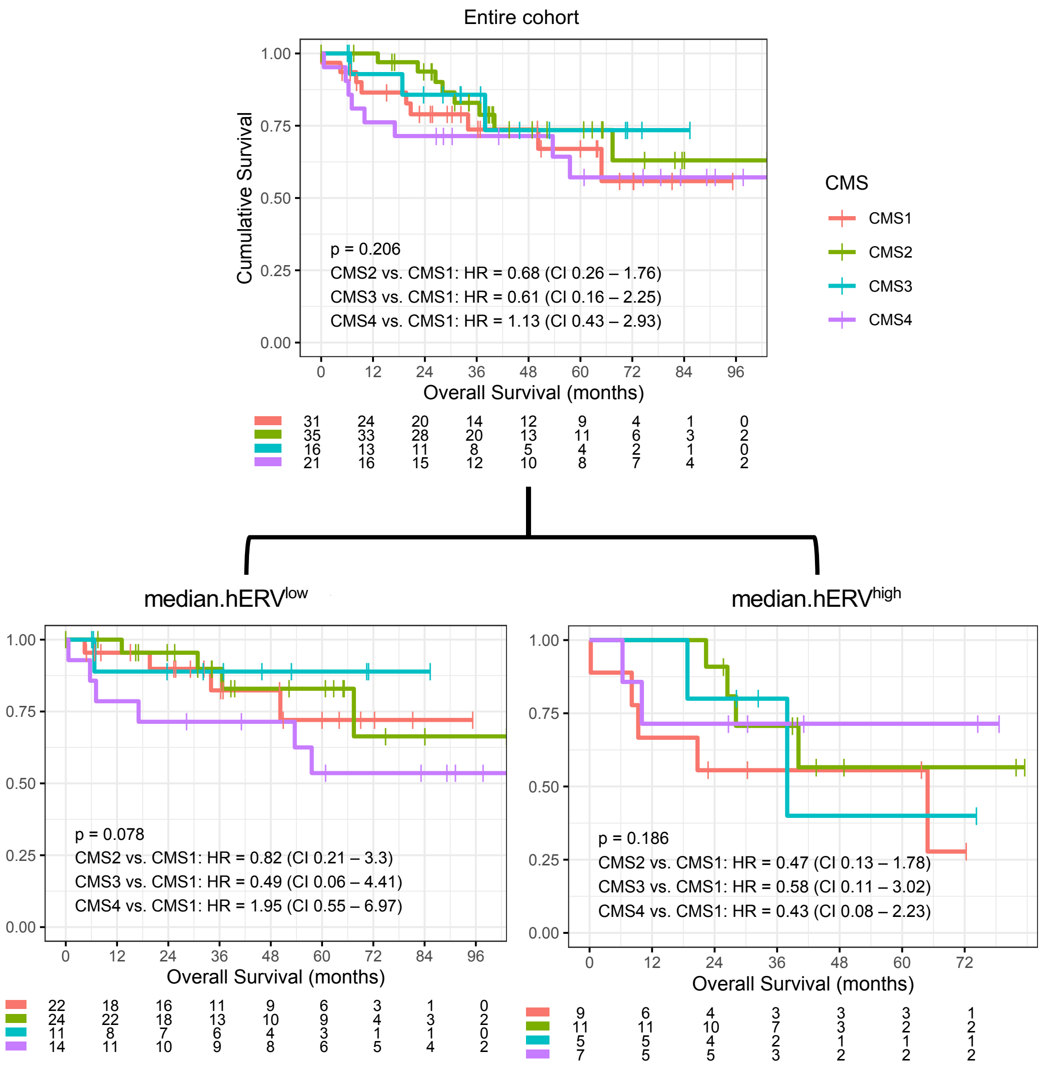

Kaplan-Meier curves of OS for each group are shown. Incorporation of median.hERV into CMS classification boosts the predictive power CMS (log-rank P values are shown).

Supplementary Fig. 12

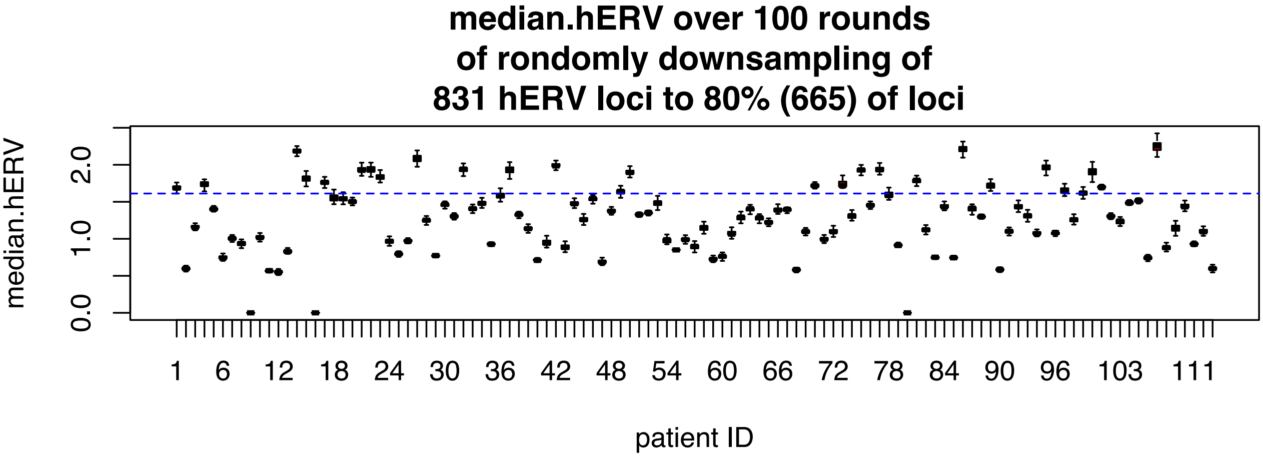

median.hERV is a robust biomarker to noise and missed hERV loci. Boxplots demonstrates only a small variation of median.hERV over 100 rounds of sampling of hERV loci down to only 80% of the originally curated reference.

Supplementary Data Legends

Supplementary Data 1: summary of WTS assay sequencing analysis metrics
Supplementary Data 2: summary of WES assay sequencing analysis metrics
Supplementary Data 3: normalized hERV expression for each patient
Supplementary Data 4: tumor infiltrating lymphocytes estimations
Supplementary Data 5: normalized gene expression values (TPM) for each patient
Supplementary Data 6: summary of all features used for survival analysis
Supplementary Data 7: summary of TSO500 assay sequencing analysis data
Supplementary Data 8: correlation between immune check point molecules and median.hERV across patients.
Supplementary Data 9: summary of all genomic and transcriptomic features included in this study for all patients.
